# Increase of scabies infestations, Norway, 2006 to 2018

**DOI:** 10.2807/1560-7917.ES.2019.24.23.190020

**Published:** 2019-06-06

**Authors:** E Amato, LS Dansie, GM Grøneng, HS Blix, H Bentele, L Veneti, P Stefanoff, E MacDonald, HH Blystad, A Soleng

**Affiliations:** 1 Department for Vaccine Preventable Diseases, Norwegian Institute of Public Health, Oslo, Norway; 2 European Centre for Disease Prevention and Control (ECDC) Fellowship Programme/EUPHEM, Stockholm, Sweden; 3 Department of Drug Statistics, Norwegian Institute of Public Health, Oslo, Norway; 4 Department of Infectious Disease Epidemiology and Modelling, Norwegian Institute of Public Health, Oslo, Norway; 5 Antibiotic Resistance and Infection Prevention, Norwegian Institute of Public Health, Oslo, Norway; 6 Department Zoonotic, Food- and Waterborne Infections, Norwegian Institute of Public Health, Oslo, Norway; 7 Tuberculosis, Blood Borne and Sexually Transmitted Infections, Norwegian Institute of Public Health, Oslo, Norway; 8 Department of Pest Control, Norwegian Institute of Public Health, Oslo, Norway

**Keywords:** Scabies, Outbreak, Treatment, Epidemiology, Syndromic surveillance, Mite infestations, Norway

## Abstract

Between October and December 2018, several clinicians in Norway reported an increase in scabies diagnoses. We compared data from the Norwegian Syndromic Surveillance System on medical consultations for mite infestations with scabies treatment sales data to investigate this reported increase. From 2013 to 2018, consultations and sales of scabies treatments had almost increased by threefold, particularly affecting young adults 15–29 years. We recommend to increase awareness among clinicians to ensure timely diagnosis and treatment.

Between October and December 2018, several general practitioners (GPs) and dermatologists reported increasing numbers of patients with scabies to the Norwegian Institute of Public Health (NIPH). In addition, the Deparment for Pest Control at NIPH received many enquiries regarding scabies from members of the public, school nurses, kindergartens and long-term care facilities. As scabies is not notifiable in Norway, we investigated alternate data sources to confirm the increase in order to identify risk groups and target control measures.

## Data sources

We extracted data on consultations from the Norwegian Syndromic Surveillance System (NorSySS) and reported outbreaks from the Norwegian disease outbreak notification system (Vesuv) from 2006 to 2018. NorSySS contains records of consultations at GP offices and out-of-hours primary care facilities in Norway. Although there is not a specific diagnosis code for scabies in the NorSySS database, the International Classification of Primary Care (ICPC-2) code ‘S72 – infestation mites’ [1] was considered the most appropriate proxy for scabies. From Vesuv, we extracted all reported outbreaks of scabies, although reporting of community-based outbreaks of scabies is not mandatory.

We compared NorSySS data with the data on drug use from the Norwegian Drug Wholesales Statistics database and the Norwegian prescription database (NorPD). Wholesales Statistics covers all sales of medicines in Norway, including over-the-counter (OTC), while NorPD includes all prescription drugs dispensed from Norwegian pharmacies. In Norway, the recommended first-line treatment for scabies is topical permethrin (5% cream). Oral ivermectin is prescribed in severe cases or in cases of treatment failure.

## Increase in mite infestations and scabies outbreaks

**Figure 1:**
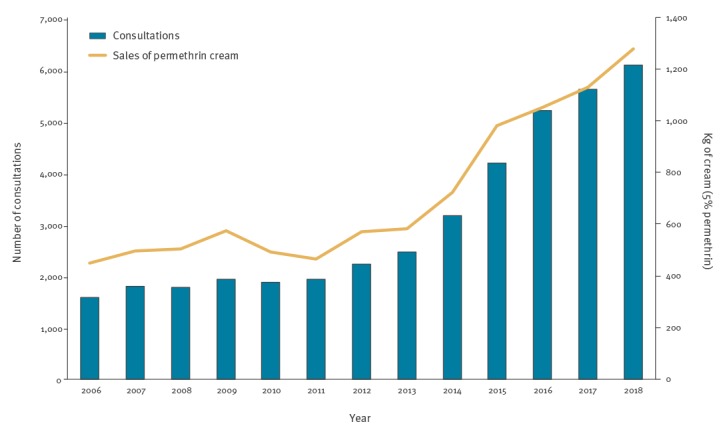
Number of mite infestation consultations vs sales of permethrin in Norway, 2006–2018

Between 2006 and 2018, 39,796 consultations for mite infestations were registered in NorSySS. Of these, 26,681 (67%) were reported from 2013 to 2018. Following an average of 1,815 consultations registered annually from 2006 to 2012, we observed an increasing trend starting in 2013, when 2,457 consultations were registered. The number of consultations for mite infestations during the study period was highest in 2018 at 6,080 consultations, which was almost threefold increase compared with 2012 (Figure 1). Moreover, we have observed that the increasing trend has continued during the first trimester of 2019, with 60% more diagnoses compared with the same period in 2018 (n = 2,423 vs n = 1,512).

**Figure 2:**
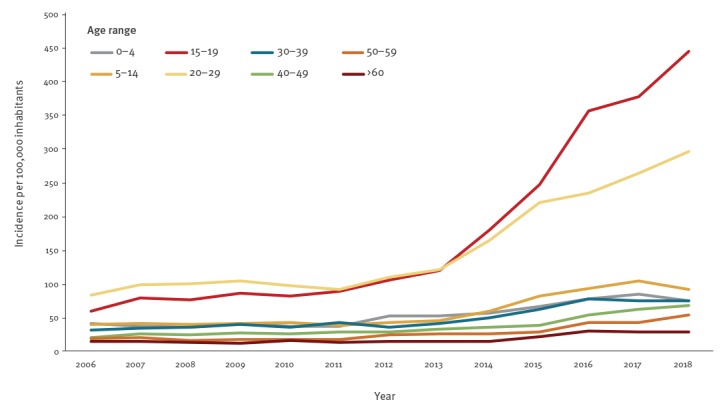
Incidence of mite infestation consultations, by age group, Norway, 2006–2018

Between 2006 and 2018, an increase in consultations for mite infestations was observed in all age groups, but the increase was most notable in those aged 15–29 years. The distribution in the age of patients was significantly different (Pearson chi-squared p value < 0.001) during 2006–12 in comparison with the period 2013–18 with a higher proportion of patients in age group 15–19 years (14% vs 21%) and 20–29 years (31% vs 34%) between 2013 and 2018. After 2012, the incidence of mite infestations notably increased in patients between 15 and 29 years old. The mean annual incidence for the age groups 15–19 years and 20–29 years increased between the periods 2006–12 (81 and 97/100,000, respectively) and 2013–18 (286 and 215/100,000, respectively) (Figure 2). In 2018, the highest incidence was reported in the age groups 15–19 years and 20–29 years (443 and 296/100,000 inhabitants, respectively) with a male:female ratio of 0.9 and 1.4. During 2006-18, mite infestations followed a seasonal pattern with most cases reported during September–November (n = 12,734, 32%) and December–February (n = 10,347, 26%), with the highest number of patients reported between October and December (week 40–49).

Seven small outbreaks, with an average of nine cases (range 2–15), were reported through Vesuv starting from 2018. Scabies outbreaks were recorded in long-term care facilities (n = 3), private households (n = 2) and other institutions (n = 2).

## Increase in sales of scabies treatments

Data from the Wholesales Statistics showed increasing sales of permethrin starting in 2014, with the largest amount of cream sold in 2018 (1,300 kg). GPs prescribed around 10% of sold permethrin and the remaining amount was distributed through over-the-counter sales. Sales of permethrin followed a similar trend as the NorSySS data during the period 2006–18 (Figure 1).

Although ivermectin was not frequently prescribed, we also observed an increasing trend in ivermectin treatment prescriptions registered in NorPD, with an increase first observed in 2012. Prescriptions then decreased until 2014 before rising rapidly to the highest observed level of sales during the study period in 2018. According to data from the Norwegian Medicines Agency (NoMA), the main indication for prescribing ivermectin since 2016 was treatment of scabies (n = 1,055 prescriptions, 73.2%) or unsuccessful use of permethrin (n = 96 prescriptions, 6.7%).

## Geographical distribution of mite infestations

**Figure 3:**
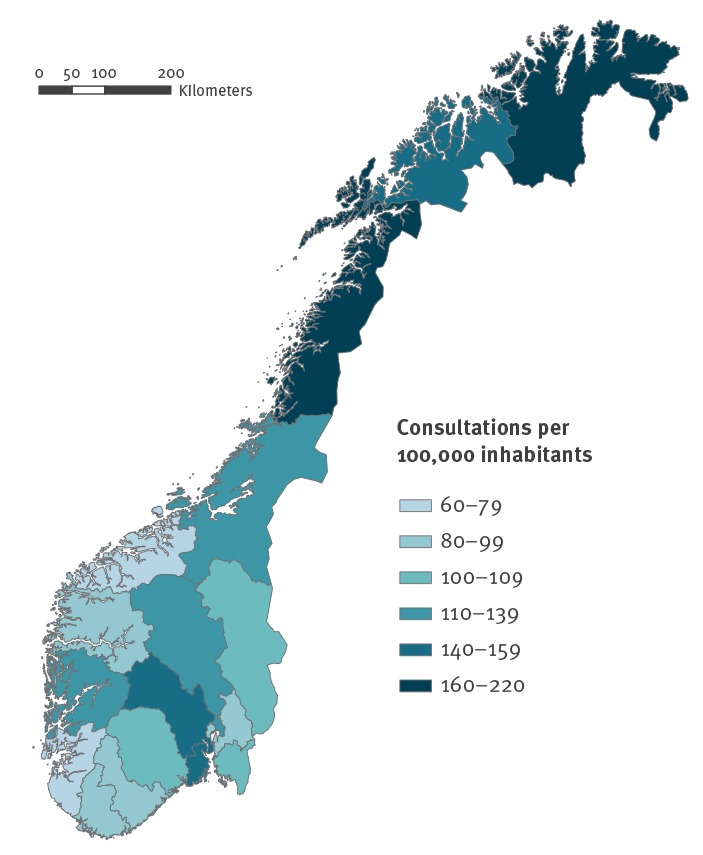
Incidence of mite infestation consultations per 100,000 inhabitants, by county, Norway, 2018

An increasing incidence of mite infestation consultations was observed in all Norwegian regions during the period 2006–18 (Table 1). In 2018, the highest incidence of consultations was reported in the northern region, followed by central, east, west and south regions with incidences of 182.3, 118.6, 116.5, 93.5 and 83.0 cases per 100,000 inhabitants, respectively (Figure 3).

**Table 1:** Incidence of mite infestation consultations per 100,000 inhabitants, by year and region, Norway, 2006–2018

Year	Region					Overall
	North	Central	West	South	East	
2006	35.4	41.8	35.9	22.5	32.5	33.9
2007	33.1	52.7	35.6	36.5	38.7	38.5
2008	44.8	48.4	31.9	41.5	36.6	37.5
2009	56.5	39.3	37.5	23.9	40.7	40.3
2010	39.7	46.2	34.4	42.0	39.0	38.7
2011	39.1	35.4	36.3	46.7	40.5	39.2
2012	36.3	51.5	47.3	58.8	41.9	44.6
2013	47.8	59.7	43.2	50.8	49.4	48.6
2014	68.2	62.3	64.8	62.6	58.8	61.8
2015	103.4	88.8	75.7	86.6	77.5	80.9
2016	141.7	96.7	97.0	113.6	92.1	99.6
2017	161.6	104.0	91.6	101.1	105.8	106.8
2018	182.3	118.6	93.5	83.0	116.5	114.8

North region: Finnmark, Troms and Nordland. Central region: Trøndelag. West region: Møre og Romsdal, Sogn og Fjordane, Hordaland and Rogaland. South region: Vest-Agder and Aust-Agder. East region: Hedmark, Oppland, Buskerud, Akershus, Oslo, Østfold, Vestfold and Telemark.

## Discussion and conclusions

Scabies is a parasitic skin infestation caused by *Sarcoptes scabiei* variant *hominis* mite that is usually spread through prolonged skin-to-skin contact. Infestation with scabies mites results in an intensly itchy skin eruption consisting of papules, nodules and vescicles [2]. Although scabies is a very common disease worldwide, the prevalence is unknown. Population-based data from Europe are lacking since scabies is not a notifiable disease in most European countries [3]. Nevertheless, the increase in consultations, along with the simultaneous increase in sales of scabies treatments and outbreaks detected in Norway, suggest an increase in incidence of scabies since 2013. In recent years, an increasing number of cases has also been reported in other European coutries, including Croatia and Germany [4,5]. The seasonal trend we observed is also consistent with other European countries where scabies is more frequently reported in wintertime as cool and moist environments increase the survival of mites [6,7].

Scabies is commonly reported in children (0–9 years old), young adults (10–19 years old) and elderly people (> 80 years old) [8]. The Norwegian data indicate few consultations (0.9%) reported among elderly people and highest proportion of consultations (58.1%) among the age groups 15–19 and 20–29 years between 2006–18. Although scabies has been more frequently described in females than males [8-11], we observed a male to female ratio of 1.4:1 in the age group 20–29 years. Further research is currently underway to explain the factors associated with transmission in these groups in Norway. In some European countries, migration and travel have been investigated as factors possibly associated with scabies [4,5,12], particularly in the context of screening of asylum seekers upon arrival and mass treatment of scabies in reception centers [13,14].

Given that scabies infestations occur after prolonged skin-to-skin contact we cannot exclude that transmission through sexual contact may also explain the increased incidence among young adults [15]. Mellanby et al. [16] showed that indirect transmission through furniture and fomites is also plausible, but it is unlikely to play a significant role unless the person is heavily infested (e.g. immunocompromised persons with crusted scabies). Additionally, we cannot exclude that the increase observed in the first trimester of 2019 could have been influenced by national media attention as this was a widely covered topic in Norway.

There are several limitations in this investigation. The reported incidence of consultations is likely to overestimate scabies infestations. First, the ICPC-2 code S72 can include symptoms caused by other types of mites. Second, the lack of microscopic detection of mites decreases the predictive value of clinical diagnosis [4]. Third, physicians can record recurrent infestations and treatment of close contacts under the same code. Data on sales of scabies treatments may also reflect the number of treatments for diagnosed scabies in addition to the number of close contact treatments.

Although we cannot yet conclude on the reasons for the demonstrated increase, clinicians should be vigilant to ensure scabies is being appropriately diagnosed and treated taking also into account that itching can persist for 2–4 weeks after the end of treatment, in some cases even longer [17]. As scabies is largely diagnosed on clinical grounds since there are no standardised laboratory tests available [2], treatment failure may occur due to wrong diagnosis, incorrect use of cream, or improper intake of oral treatments [4]. Therefore, early diagnosis confirmations and proper treatment are critical for avoiding the spread of infestation and outbreaks [18].
